# Metal–Organic Framework Gels for Adsorption and Catalytic Detoxification of Chemical Warfare Agents: A Review

**DOI:** 10.3390/gels9100815

**Published:** 2023-10-13

**Authors:** Ye Zhang, Cheng-An Tao

**Affiliations:** College of Science, National University of Defense Technology, Changsha 410073, China; zhangye8905@foxmail.com

**Keywords:** metal–organic framework, gel, chemical warfare agents, catalytic detoxification

## Abstract

Chemical warfare agents (CWAs) have brought great threats to human life and social stability, and it is critical to investigate protective materials. MOF (metal–organic framework) gels are a class with an extended MOF architecture that are mainly formed using metal–ligand coordination as an effective force to drive gelation, and these gels combine the unique characteristics of MOFs and organic gel materials. They have the advantages of a hierarchically porous structure, a large specific surface area, machinable block structures and rich metal active sites, which inherently meet the requirements for adsorption and catalytic detoxification of CWAs. A series of advances have been made in the adsorption and catalytic detoxification of MOF gels as chemical warfare agents; however, overall, they are still in their infancy. This review briefly introduces the latest advances in MOF gels, including pure MOF gels and MOF composite gels, and discusses the application of MOF gels in the adsorption and catalytic detoxification of CWAs. Meanwhile, the influence of microstructures (pore structures, metal active site, etc.) on the detoxification performance of protective materials is also discussed, which is of great significance in the exploration of high-efficiency protective materials. Finally, the review looks ahead to next priorities. Hopefully, this review can inspire more and more researchers to enrich the performance of MOF gels for applications in chemical protection and other purification and detoxification processes.

## 1. Introduction

**Figure 1:**
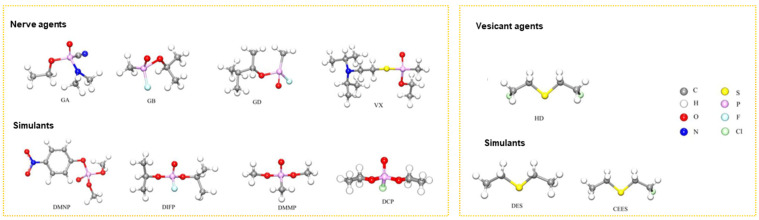
Structure of typical CWAs and simulants [11]. Copyright © 2023, Elsevier.

Chemical warfare agents (CWAs) are toxic chemicals employed in warfare or related military operations to harm, kill, or paralyze adversaries. Nerve agents and vesicant agents are the most lethal types of chemical warfare agents [1]. Nerve agents are derived from alkyl phosphonate esters, which can cause neurological disorders, damage the nervous regulatory system and respiration processes, and lead to suffocation within minutes. Common nerve agents include tabun (GA), sarin (GB), soman (GD), and VX (left, Figure 1) [2,3]. Vesicant agents can cause severe skin erosion and damage to the respiratory and digestive tracts and have systemic toxic effects, potentially leading to death. Mustard gas (HD) is a commonly used vesicant agent [4,5,6]. Although chemical weapons are regulated by the Chemical Weapons Convention, the potential for their use by extremist countries or organizations remains. Therefore, the development of effective protective materials against chemical warfare agents remains crucial [7,8,9,10,11]. Due to the highly toxic nature of nerve and erosive agents, their simulants with lower toxicities (right, Figure 1) are often used in research to reduce the risk of accidental poisoning.

Currently, activated carbon is the primary material used for protection against chemical warfare agents. It functions by adsorbing toxic substances, and, in some cases, it can be impregnated with additional substances to enhance its catalytic degradation capabilities, converting CWAs into non-toxic compounds [12]. However, activated carbon materials suffer from several limitations, including a low adsorption capacity, a limited number of active sites, susceptibility to inactivation or destruction of catalytic sites, slow reaction kinetics, and poor structural flexibility.

In recent years, there has been a focus on developing fast, simple, safe, and effective detoxification methods for chemical warfare agents. Scientists have explored various materials with good catalytic performance to achieve better detoxification results [13,14,15,16,17]. These materials typically possess specific structural characteristics, such as larger pore sizes, higher specific surface areas, and flexible structures that provide more active sites. Continued research has led to the discovery of catalytic materials that exhibit excellent performance in the degradation of CWAs, including metal oxides, polyoxometalates, and metal clusters [18,19,20,21]. For instance, Wang et al. designed a composite conjugated microporous polymer based on Fe^2+^ for detoxification purposes [22]. Hu et al. developed recoverable amphiphilic polyoxoniobates that catalyze oxidative and hydrolytic decontamination of chemical warfare agents [23]. Zang et al. prepared porphyrinic silver cluster assembled materials for simultaneous capture and photocatalysis of mustard gas simulants [24].

**Figure 2:**
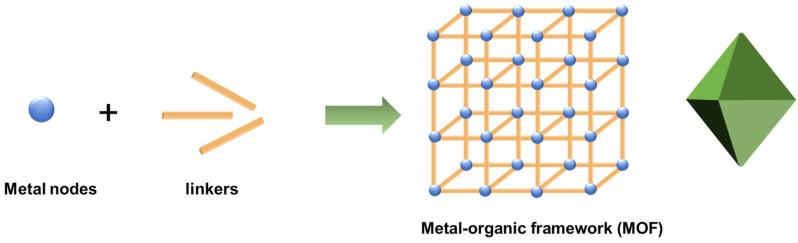
The formation of MOFs.

Among the numerous detoxification materials, metal–organic frameworks (MOFs) formed by metal ions or clusters and multidentate ligands have received widespread attention (see Figure 2) [11,25,26,27,28,29,30]. The metal ions or clusters are mainly derived from transition metal and lanthanide salts, and the multidentate ligands include bridging carboxylic acids, imidazole, porphyrin, etc. Various methods are used to prepare MOFs, and hydrothermal and solvothermal approaches at low temperatures (<250 °C) are the most commonly used at the early stage. Other common methods, such as microwave synthesis and ultrasonic synthesis, have been developed at present for large-scale synthesis, rapid reaction, and reduction of crystallites size. MOFs have an inherently large specific surface area and abundant pore structure, which give them excellent adsorption or solid-phase extraction properties [31,32,33,34,35]. Moreover, metal nodes in MOFs serve as Lewis acid catalytic active centers, which promote the hydrolysis of chemical warfare agents [36]. The photoactive linkers may serve as photocatalysts [37,38]. MOF materials are usually in powdered crystalline states and have poor processability. Agglomerated particles may lead to a decrease in active sites, limiting their practical applications. These problems pose a challenge for practical applications. 

MOF (metal–organic framework) gels are a class with an extended MOF architecture that are mainly formed using the metal–ligand coordination as effective force to drive gelation, and these gels combine the unique characteristics of MOFs and organic gel materials [39]. They have the advantages of a hierarchically porous structure, large specific surface area, machinable block structures and rich metal active sites. MOF gels also easily form bulk materials and can be shaped as needed [40]. These materials not only overcome the limitations of MOF powders in practical applications but also contribute to reduce the diffusion barrier between the matrix and active sites, accelerate the mass transfer rate, and enhance the adsorption and catalytic performance [40,41]. MOF gels can be used as adsorbents for removal of hazardous heavy metal ions and organics in water and to capture harmful gases and eliminate particulate matters, such as PM2.5 and PM10. The catalytic applications of MOF gels include electrocatalysis for fuel cells, heterogeneous catalysis for organic chemistry, and photocatalysis for removal of pollutants. The unique structures and composition of MOF gels also inherently meet the requirements for adsorption and catalytic detoxification of CWAs. Metal organic composite aerogels have the advantages of aerogels, showing low density, a high specific surface area and a multistage pore structure, which is conducive to the transport of toxic molecules and degradation of products in the aerogels. It is also possible to retain metal oxygen cluster nodes through a reasonable design and disperse the metal nodes through appropriate organic molecules to ensure catalytic activity, and many related works have been reported. Recently, there have been a few reviews on MOF gels [42,43,44]; however, research on the application of MOF gels for the adsorption and degradation of CWAs lacks a systematic introduction and review.

In this review, pure MOF gels containing different metal ions and the formation process are introduced first. Then, the recent progress of MOF gel composites is summarized. The application of these MOF gel composite materials to protect against nerve agent and vesicant agent CWAs is also discussed. It also looks forward to the next research focuses on the use of MOF gels for CWA protection. The results of this paper provide new ideas for the research and development of novel efficient protective materials.

## 2. Pure Metal–Organic Framework Gel

**Figure 3:**
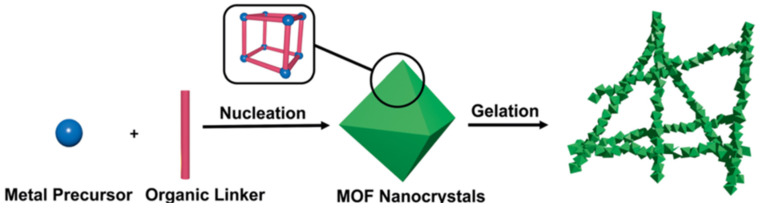
Schematic representation of pristine MOG formation. Reproduced from Ref. [43] with permission from the Royal Society of Chemistry.

Pure MOF gels refer to gel materials consisting of a single MOF material, including xerogel and aerogel, that served as the backbone structure. Pure MOF gels are usually synthesized by directly mixing the metal precursor and organic linker, and the formation process is simple and controllable (see Figure 3) [40,43]. When the coordination polymer separates from the solvent and prevents the solvent from flowing, the gels are obtained. The porous aerogels formed after post-processing have rigid spongy network that consist of nanometer-sized MOF particles. At present, metals with different valence states have been successfully used to prepare MOF gels, typical of which are tetravalent Zr(IV), trivalent Fe(III), Al(III), Cr(III), bivalent Zn(II) and Cu(II), monovalent Ag(I) [45,46,47,48,49,50,51]. MOF gels containing a variety of metals have also been prepared [52,53]. 

### 2.1. Metal(IV)-MOF Gels

Many early reports describe gel formation during the synthesis of UiO-66, a typical zirconium(IV)-carboxylate MOF [54,55,56]. For example, Liu et al. reported Zr-MOF gel synthesized from an ethanol–DMF mixture containing aminoterephthalic acid and ZrCl_4_ [57]. Then, Bueken et al. first reported hierarchically porous, monolithic Zr-MOF xero- and aerogels consisting of several prototypical Zr^4+^-based MOF nanoparticles, including UiO-66-X (X = H, NH_2_, NO_2_, (OH)_2_), UiO-67, MOF-801, MOF-808, and NU-1000 [58]. Among them, the UiO-66 xerogel has a BET surface area of 1459 m^2^·g^−1^, and the total pore volume was 2.09 cm^3^·g^−1^, higher than that of bulk UiO-66 powder. 

**Figure 4:**
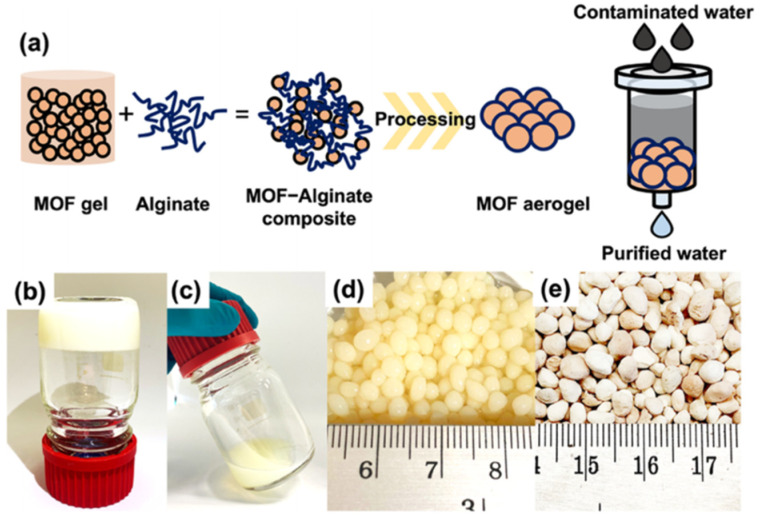
(**a**) Design concept of MOF aerogel for use in decontamination of arsenic species in water. Optical images of UiO-66-NH_2_ in the formation of (**b**) a nonflowing gel, (**c**) fluid gel, (**d**) hydrogel, and (**e**) aerogel. Reprinted with permission from [59]. Copyright 2022 American Chemical Society.

Moreover, as shown in Figure 4, UiO-66-NH_2_ aerogel has been designed as an efficient adsorbent for the trace adsorption of arsenic in water in the full pH range (pH 1–14) [59]. These aerogel have advantages in terms of processability and preventing back pressure during the continuous flow process compared with pristine UiO-66.

### 2.2. Metal(III)-MOF Gels

MOF gels for trivalent metals are the most widely studied, of which iron(III)-based MOF gels were the first to be synthesized. Martin R and coworkers firstly reported metal–organic framework aerogels that were synthesized by mixing Fe(NO_3_)_3_ and trimesic acid in 2009 [60]. The resultant gels have an elemental formula similar to that of MIL-100(Fe)(Fe_3_O(BTC)_2_F·2H_2_O) and possess high internal micro- and macroporosity. Their specific surface area and total pore volume can reach as high as 1618 m^2^·g^−1^ and 5.62 cm^3^·g^−1^, respectively. To date, most of the functional pure Fe-MOF gels are still formed by Fe^3+^ and carboxylic acids, especially 1,3,5-benzenetricarboxylic acid (BTC). For example, Hu et al. developed Fe^3+^–(BTC) metal–organic hybrid gel for online enrichment of trace analytes in a capillary [61]. Zheng et al. synthesized monolithic MIL-100(Fe) with 1,3,5-benzenetricarboxylic acid for energy-efficient removal and recovery of aromatic volatile organic compounds [46]. 

**Figure 5:**
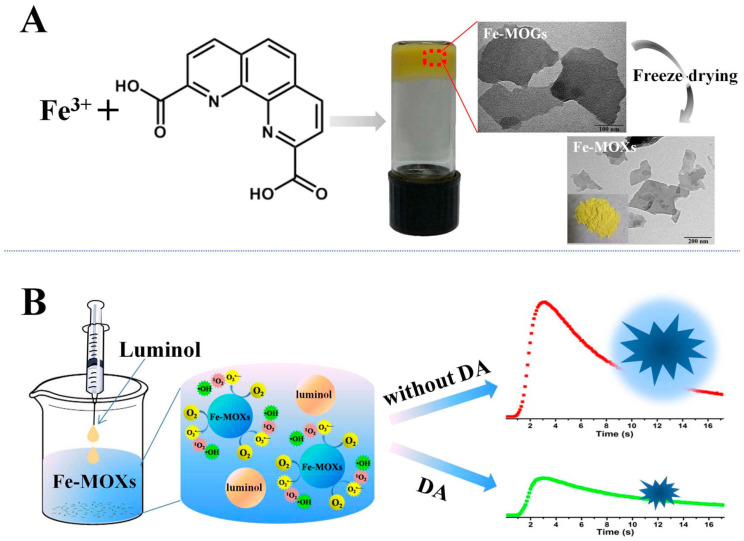
Schematics To Show the Preparation of Fe–MOGs (**A**) and the CL Detection of DA with Fe–MOXs (**B**). Reprinted with permission from [63]. Copyright 2017 American Chemical Society.

Su and coworkers reported a series of porous Fe-MOF aerogels produced from Fe^3+^ and bridging carboxylic acids [62] and revealed a simple formation mechanism. The porous aerogels were prepared using three steps: (1) primary nanoparticles were formed via Fe-carboxylate coordination; (2) primary nanoparticles condense together to form networks with an open, continuous and porous structure; and (3) the porous aerogels are produced after a subcritical CO_2_(l) extraction process. The highly porous aerogels can be prepared when rigid bridging carboxylates were used, such as 1,4-benzenedicarboxylate, which possesses a higher of BET surface area of 1454 m^2^·g^−1^. A sensitive detection method of dopamine (DA) was proposed given that DA greatly inhibits the Fe-MOX-catalyzed luminol CL (see Figure 5), representing the first example of the use of MOF gels as catalysts for a sensing platform in the CL field [63].

**Figure 6:**
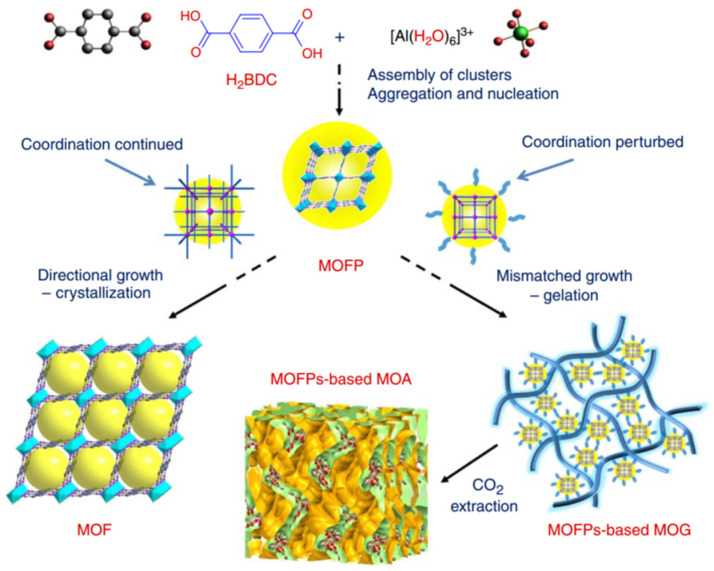
Schematic representation of the formation of MIL-53(Al) MOF versus MOF aerogel [47]. Copyright © 2013, The Authors.

Al(III)-based MOF gels have also gained attention. Su et al. prepared gel electrolytes that have a sponge-like porous matrix of metal–organic gel assembled by coordination of Al^3+^ and 1,3,5-benzenetricarboxylate (H_3_BTC) for use in highly efficient quasi-solid-state dye-sensitized solar cells (DSSCs) for the first time [64]. Then, a variety of ultralight hierarchically micro/mesoporous Al-MOF aerogels were also first successfully synthesized by Su et al. [47]. As shown in Figure 6, these aerogels are formed through the stepwise assembly of light metal Al(III) with bridging carboxylic acids. In the early stage, the metal ions and ligands assemble into an MOF cluster, which can polymerize or aggregate to trigger nucleation, and the nucleation of new particles is retarded as the concentrations of ligands and metal ions decrease. Then, the consistent epitaxial growth or oriented attachment induced by surface intension will lead to the crystallization of bulky MOFs when the conditions favor the crystal growth of the precursors. However, if the coordination equilibria are perturbed by other competing interactions, non-crystallographic branching may occur, leading to mismatched growth or cross-linking, which provide the opportunity for gelation. The final Al-MOF aerogels were obtained after the careful removal of solvents via sub/supercritical CO_2_ extraction.

In addition to the above reported MOF gels containing Al(III) and Fe(III), Cr(III) ions can also be used for preparing MOF gels. Su et al. reported on MOF aerogels based on Cr^3+^ and bridging carboxylic acids [62]. Heating induces the formation of these Cr(III)-carboxylate gels, and all of the Cr^3+^-containing gels could only be formed at temperatures above 80 ℃. The texture and porosity of the aerogels are affected by the reactant concentration and organic ligands. At high reactant concentrations (Cr:BDC = 2:3, 0.2 mol·L^−1^), the Cr-BDC aerogel has a high BET surface area of 737 m^2^·g^−1^. 

### 2.3. Metal(II)-MOF Gels

Lee and coworkers developed a luminescent Zn-MOF hydrogel that achieved high sensitivity detection of TNT [65]. Tian and coworkers reported monolithic HKUST-1(Cu-MOFs) with higher volumetric BET areas (1193 m^2^/g), pore volumes (0.52 cm^3^·g^−1^), and adsorption capacities compared to traditional powdered counterparts [50]. It also has a high bulk density of 1.06 g cm^−3^ and exhibits enhanced methane uptake of 259 cm^3^ (STP) cm^−3^ at 65 bar.

### 2.4. Metal(I)-MOF Gels

Su et al. also reported luminescent coordination polymer gels based on rigid terpyridyl phosphine and Ag(I), and the terpyridine groups could generate interesting photochemical and electronic properties. The gel emits blue luminescence that exhibits an emission intensity comparable with that of the ligand in dilute solution [51]. 

Cheng et al. synthesized a silver(I) coordination polymeric gelator through the combination of Ag(I) and 2, 7-bis(1-imidazole) fluorene. This coordination polymeric gel exhibited thixotropic behaviors and stimuli responsive to S^2−^, I^−^ and displayed antibacterial properties [66].

### 2.5. Multi-Metal-MOF Gels

A series of bimetallic Co/Fe-MOF xerogels that have sufficient adsorption sites for CO_2_ molecules have been prepared, and the metal center of Co acts as a major active site for photocatalysis [67]. This novel bimetallic xerogel exhibits enhanced adsorption and utilization of light energy and improved separation and transfer of carriers. Therefore, the conversion of CO_2_ to CO is rapidly promoted, and the Co/Fe xerogel exhibits a high CO yield (67 μmol g^−1^ h^−1^) when the mole ratio of Co: Fe was set to 1:3, far higher than that of the single Fe center MOF xerogel.

## 3. Metal–Organic Framework Composite Gel

The formation of pure MOF gels is affected by reaction conditions, such as reactant concentration ratios, temperature, and their structures and applications are limited. The metal–organic framework composite gels formed by growing or aggregating MOF particles into interconnected 3D networks, such as cellulose, graphene, silicon aerogels, etc., exhibit various architectures and are useful in a variety of applications. The MOF composite gels are mainly based on Zr-MOFs, Fe-MOFs, Cu-MOFs, Co-MOFs, and others.

### 3.1. Zr-MOF Composite Gel

**Figure 7:**
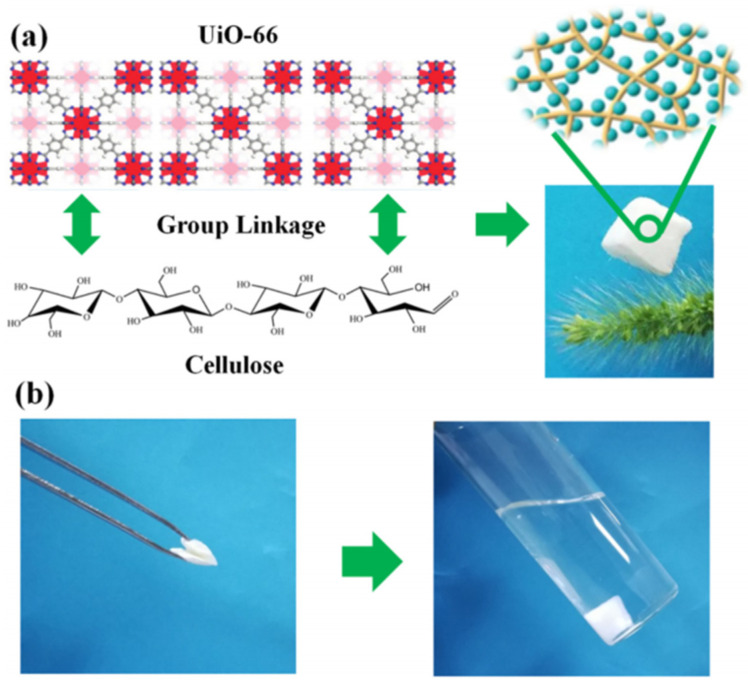
The fabrication process of the MOF/NC aerogel and photographs of the lightweight MOF/NC (**a**). Photographs show the deformed MOF/NC would recover its original shape when put back to solution (**b**) [73]. Copyright © 2019, Elsevier.

Zr-MOF is one of the most stable MOFs, and many researchers are committed to fabricating Zr-MOF composite gels with other skeleton materials. Currently, Zr-MOF composite gels containing UiO-66 have been most widely studied [45,68,69,70,71]. The UiO-66 nanoparticles can still retain their crystallinity and function when integrated within various substrates, such as cellulose nanocrystal (CNC) aerogels, and the obtained flexible and porous composite gels show good processability [72]. The oxygen-containing groups on UiO-66 (Zr-OH) are physically crosslinked with the hydroxyl groups in cellulose by hydrogen bonding. As shown in Figure 7, UiO-66/NC was obtained using nanocellulose as the structural skeleton. This composite gel has a specific surface area of 826 m^2^ g^−1^ and can stand on the bristle of grass without observable deformation [73]. 

In addition, there are also several studies on other Zr MOF composite gels, such as NU-1000. The gel is formed by grafting NU-1000 into agarose (AG) possessing micropores, mesopores, and macropores, and the average pore size is 2.57 nm, which is close to that of NU-1000. This hybrid aerogel has potential applications for adsorbing in water treatment due to the hierarchical pore structure [70].

### 3.2. Fe-MOF Composite Gel

**Figure 8:**
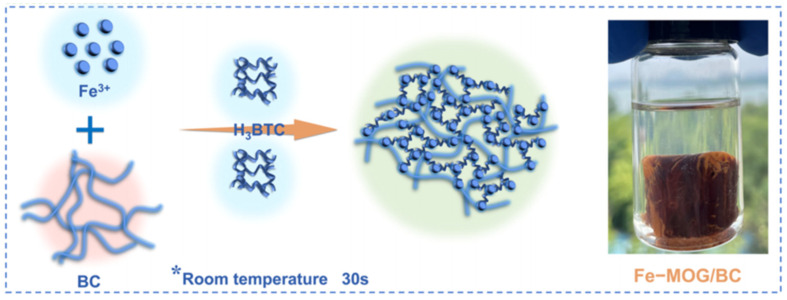
Schematic Illustration of the Fabrication Process of the Fe-MOG/BC Aerogel. Reprinted with permission from [74]. Copyright 2021 American Chemical Society. *: the formation conditions of gel.

At present, the Fe-MOF gel is one of the most studied pure MOF gels, and it also attracts much attention for the fabrication of composite gels. Researcher have successfully constructed Fe-MOF composite gels with many suitable porous supports, such as cellulose, graphene, aerogels, etc. As shown in Figure 8, a monolithic iron metal−organic gel/bacterial cellulose (denoted as Fe-MOG/BC) composite has been prepared by the crosslinking of nanoscale Fe-BTC MOG particles with BC nanofibers to form 3D porous networks [74]. These Fe-MOG/BC aerogel possesses many unique structural characteristics, such as a three-dimensional (3D) hierarchically porous microstructure, abundant active sites, and ultralight, water-fast, and mechanically robust features. Therefore, they exhibit a superb saturated sorption capacity (495 mg g^−1^) for arsenate, higher than that of Fe-MOF/BC.

The MOF/GA composites can be prepared using growth-oriented MIL-88-Fe synergized with graphene aerogels (GAs), and the oriented composite can be used for high-performance supercapacitors with a specific capacitance as high as 353 F g^−1^ at a scan rate of 20 A g^−1^ [75]. By immobilizing Fe-MOFs on nanofibrous aerogel membranes (NFAMs), a novel Fe-BTC@polyacrylonitrile (PAN)NFAM catalyst was constructed with a 3D interconnected hierarchical porous structure that could be used as a catalytic membrane in a filtration device for the treatment of organic wastewaters [76]. Specially, the combination of Fe-MOFs with a photocatalyst, such as g-C_3_N_4_, can enhance the visible-light adsorption regions, increase the specific surface areas and prolong the lifetime of the charge carriers. Therefore, the porous g-C_3_N_4_/NH_2_-MIL-53(Fe) aerogel showed excellent recyclability and a higher photocatalytic performance than pure g-C_3_N_4_ nanosheets [77]. 

### 3.3. Cu-MOF Composite Gel

**Figure 9:**
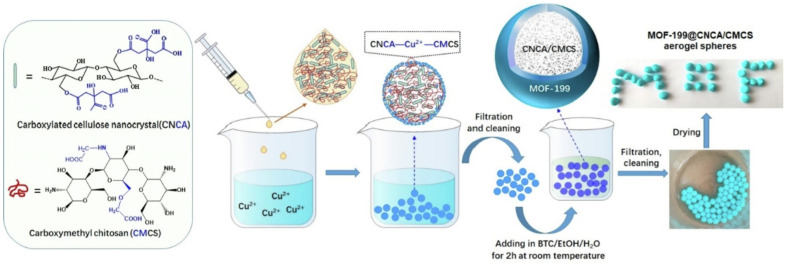
Schematic illustration of the synthetic process of MOF-199@CNCA/CMCS aerogel spheres [85]. Copyright © 2022, Elsevier.

HKUST-1, which is also called Cu-BTC and consists of copper oxide clusters linked by benzene-1, 3, 5-tricarboxylate ligands, is a common material for Cu-MOF composite gels [78,79]. HKUST-1/graphene aerogels, HKUST-1 modified ultrastability cellulose/chitosan composite aerogels, and HKUST-1 silica aerogel composites have been fabricated successfully [78,79,80,81,82,83,84]. For example, a core–shell hybrid aerogel sphere material containing Cu-MOF was fabricated using a combined assembly strategy of coordination bonding and ionic cross-linking [85]. The Cu^2+^ ions cross-linked carboxylated cellulose nanocrystals (CNCA) and carboxymethyl chitosan (CMCS) hydrogel spheres to serve as templates for the in situ growth of the MOF-199 crystal using 1,3,5-benzenetricarboxylic acid as ligand (Figure 9). The resultant aerogel spheres showed an excellent adsorption capacity towards methylene blue (MB) with values as high as 1112.2 mg·g^−1^.

**Figure 10:**
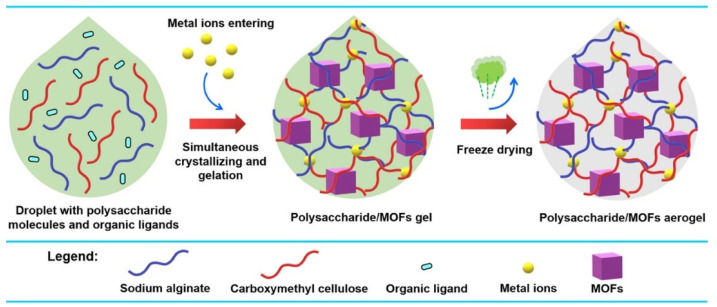
Illustration of the One-Droplet Synthesis of Polysaccharide/MOF Aerogels. Reprinted with permission from [86]. Copyright 2023 American Chemical Society.

Meanwhile, various methods are used for the synthesis of Cu-MOF composite gel. As shown in Figure 10, a one-droplet synthesis strategy was developed to synthesize functional polysaccharide/MOF(HKUST-1) aerogels [86]. In this one-droplet reaction, the metal ions initiate the cross-linking of polysaccharide molecules and coordinate with organic ligands to form MOFs simultaneously. The resulting composite aerogel has a hierarchical porous structure and exhibits a high adsorption capacity for CO_2_.

### 3.4. Co-MOF Composite Gel

**Figure 11:**
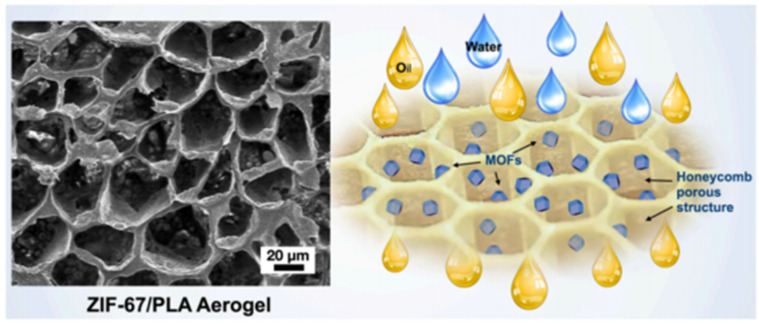
Images of the ZIF-67@PLA honeycomb aerogel structure and the oil–water separation [89]. Copyright © 2022, Elsevier.

Most of the Co-MOF composite gels consist of a zeolitic imidazolate framework-67 (ZIF-67) that is formed by 2-methylimidazole [87,88,89,90], and their structures are diverse. As shown in Figure 11, the highly hydrophobic ZIF-67@PLA honeycomb aerogel was formed by physically combining ZIF-67 nanoparticles with a PLA solution and a water-assisted heat-induced phase. These ZIF-67@PLA honeycomb aerogels have a multilayer porous structure, a considerably reduced pore size, and an increased honeycomb pore volume and exhibit better oil wettability than pure PLA aerogels [89]. 

Wood aerogels made from naturally lightweight, high-porosity, thin-walled balsa wood have a lamellar structure and provide sufficient attachment sites for ZIF-67. ZIF-67@WA (wood aerogel) has been prepared successfully through in situ anchoring of ZIF-67 on the wood aerogel, and it exhibits excellent adsorption performance for tetracycline and Cu(II) ions, respectively [91,92]. In addition, Co^2+^ coordinates with the oxygen-containing functional groups of MXene to form a hydrogel and then acts as a nucleation site for the in situ growth of ZIF-67 particles [93]. Porous 3D rGO/ZIF-67 aerogel was prepared via the assembly of ZIF-67 polyhedrons on the 3D rGO framework, which has a specific surface area up to 491 m^2^·g^−1^ and displays excellent adsorption for organic dyes [94]. 

### 3.5. Other MOF Composite Gels

In addition to the above-mentioned MOF composite aerogels, other reported MOF composite aerogels are mainly based on ZIF-8(Zn-MOF) and MIL 101(Cr-MOF). For example, nanocellulose can also serve as template for developing shapeable fibrous ZIF-8 aerogels, which exhibit higher adsorption capacity and rapid adsorption kinetics for different organic dyes [72].

**Figure 12:**
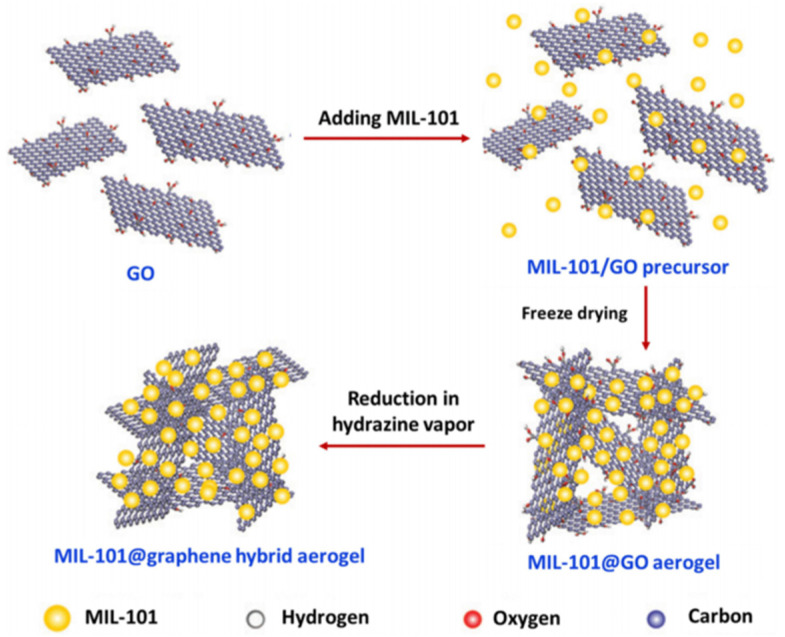
Synthesis of MIL-101@graphene hybrid aerogels. Reproduced from Ref. [95] with permission from the Royal Society of Chemistry.

As shown in Figure 12, the graphene aerogel (GA)-supported MIL-101 (Cr-MOF) particles exhibited a three-dimensional (3D) architecture with an interconnected macroporous framework of graphene sheets and uniform dispersion of MOF particles, which could be used as adsorbents for the solid-phase extraction (SPE) of non-steroidal anti-inflammatory drugs (NSAIDs) [95].

Specifically, a superhydrophobic aerogel was constructed by fine-tuning the hydrophobicity of MOF (MOF, Eu-bdo-COOH, H_4_bdo = 2,5-bis(3,5-dicarboxylphenyl)-1,3,4-oxadiazole) microspheres, and this aerogel exhibits fast and efficient absorption of various oily substances from water [96]. 

## 4. Adsorption of CWAs

### 4.1. MOFs for Adsorption of CWAs 

**Figure 13:**
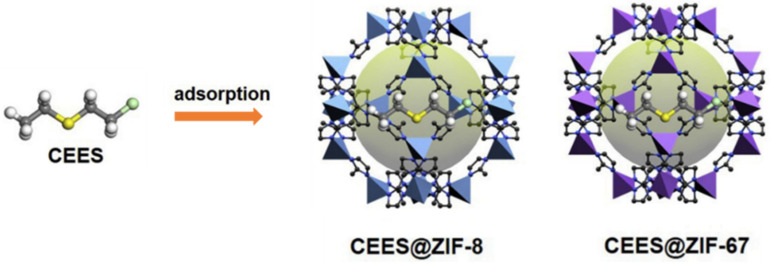
Schematic illustration of CEES adsorption by ZIF-8 and ZIF-67 [98]. Copyright © 2019, Elsevier.

The adsorptive removal of CWAs is an important method of personal protection, and effective adsorbents, such as activated carbons, metal oxides, etc., have been widely explored. A variety of studies have indicated that MOFs are promising materials for the capture of CWAs owing to their high porosity and adjustable reactivity. The selective adsorption of organic phosphonates in MOF-5/IRMOF-1 was investigated first, and the binding energy of DMMP in IRMOF1 was ∼19 kcal/mol. The sorption capacity of the CWA simulant DMMP (dimethylmethyl phosphonate) can reach as high as 0.95 g g^−1^ [97]. Both zeolitic imidazolate frameworks ZIF-8 and ZIF-67 have large pores connected through small apertures, and the inner pores exhibit strong hydrophobicity. Therefore, they exhibit excellent performance for rapid adsorption and removal of hydrophobic CEES molecules (Figure 13). The maximum adsorption capacities of ZIF-8 and ZIF-67 for CEES were 456.61 mg g^−1^ and 463.30 mg g^−1^, respectively, and 100% of HD from from the water/ethanol solution (9:1, *v*/*v*) was removed within 1 min in further experiments [98]. Some research suggests that the partial charge of the metal atom induces a higher affinity of CWAs toward the MOF surface [99]. 

Recently, zirconium-based MOFs have been extensively studied for the adsorption of chemical warfare agents (CWAs) and their simulants [100]. For example, NU-1000 and UiO-67 have been successfully used for capturing chemical warfare agent simulants 2-CEES and DMMP from aqueous media [101]. NU-1000 showed adsorptive capacities of 4.197 and 1.70 mmol g^−1^ for 2-CEES and DMMP, respectively, higher than the results of UiO-67, which can also adsorb 2-CEES and DMMP with capacities of 4.000 and 0.90 mmol g^−1^, respectively. Zr-MOFs with different surface area/pore volumes, secondary building unit (SBU) connectivity, pore functionalization, and open metal sites for the adsorption of sarin gas and CEES have been examined, and the findings showed that UiO-66, defective UiO-66, and MOF-808 have the highest reactivities toward GB due to the presence of more active sites per unit volume [102].

### 4.2. MOF Gels for Adsorption of CWAs

Currently, MOF gels containing MOF structures also demonstrate outstanding adsorption properties for CWAs or simulants. We and collaborators prepared granular UiO-66-NH_2_ xerogels that showed an excellent adsorption capacity of 802 mg/g for CEES vapor in static adsorption and desorption tests, higher than that of many active inorganic nanomaterials [103]. The ability to retain adsorbed CWA on the surface/in the porous structure is a very important feature of protection materials. Static desorption tests monitored their weight change after exposure to 2-CEES vapors for 1 day, and air desorption tests were conducted at 2 d and 7 d. The results demonstrated that these Zr-MOF xerogels have low desorption capacity with only 28 wt%. Moreover, the superelastic hierarchical aerogels composed of MOF-808 and SiO_2_ nanofibers exhibited hierarchical cellular architectures with interconnected channels. Simultaneously, the additional ceramic constituents in the interconnected channels can generate van der Waals barriers, which are beneficial for nerve agent adsorption in open MOF sites. Therefore, this MOF gel showed efficient adsorption performance against CWAs with a breakthrough extent of 400 L g^−1^ [104].

## 5. Catalytic Detoxification of CWA

According to recent reports, MOF gels have shown excellent performance in the field of CWA detoxification due to their large specific surface area, hierarchical porous structures and processability. The meso- and macropores facilitate the transport of toxic molecules and degradation products within the gel monoliths. Some substrates introduced for constructing MOF composite gels can adsorb CWAs and exhibit water storage abilities, which promote the degradation process.

Among the numerous chemical agents, nerve agents and vesicant agents are the focus of current study, and Zr-MOFs gels are the most commonly reported materials. The properties of pure zirconium-based MOF gels and zirconium-based MOF composite gels for the degradation of these two CWAs and other simulants are summarized, as shown in Table 1, and details of the analysis are presented below.

### 5.1. Nerve Agents and Simulants

**Figure 14:**
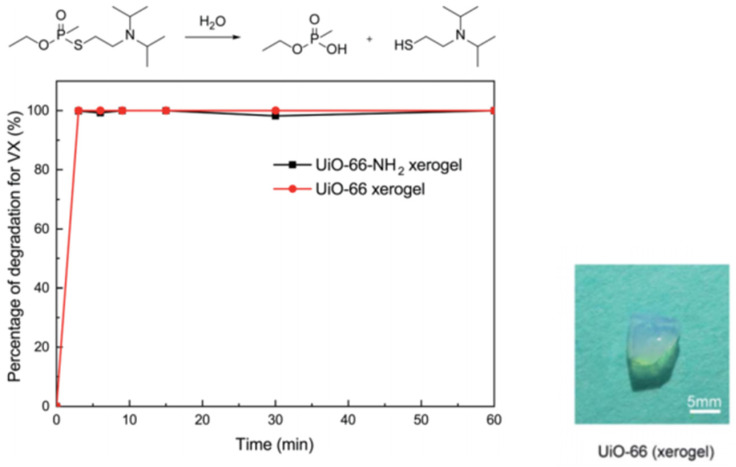
Degradation of VX on the UiO-66 xerogel and the UiO-66-NH_2_ xerogel. Reproduced from Ref. [105] with permission from the Royal Society of Chemistry.

For nerve agents and simulants, Zr-MOF gels based on UIO-66 and MOF-66 have been extensively studied. As shown in Figure 14, our group and coworkers firstly reported pure macroscopic monolithic UiO-66 and UiO-66-NH_2_ xerogels with excellent degradability for real nerve agent VX, and both of them possess a short half-life of 1.5 min and 100% conversion within 3 min. These materials can selectively catalyze the breakage of P-S during VX hydrolysis, and less toxic product breakage was obtained [105].

Sui and our group fabricated flexible UiO-66-NH_2_-loaded cellulose sponge composites for rapid degradation of DMNP. The surprising hydrolysis rate with a half-life of only 9 min was attributed to the preserved catalytic activity of MOFs and the high porosity and random three-dimensional structures of the sponge [106]. UiO-66/nanocellulose aerogel composite fabricated by simple blending of UiO-66 and TEMPO-oxidized cellulose nanofibers could decompose nearly all MPO within 3 min and exhibited a 0.7 min half-life under static condition [107]. Moreover, this aerogel composite exhibits the surprising ability to dispose 53.7 g of MPO per hour with 1 m^2^ of the effective area when used as the detoxification filter in continuous dynamic continuous flow systems. 

**Figure 15:**
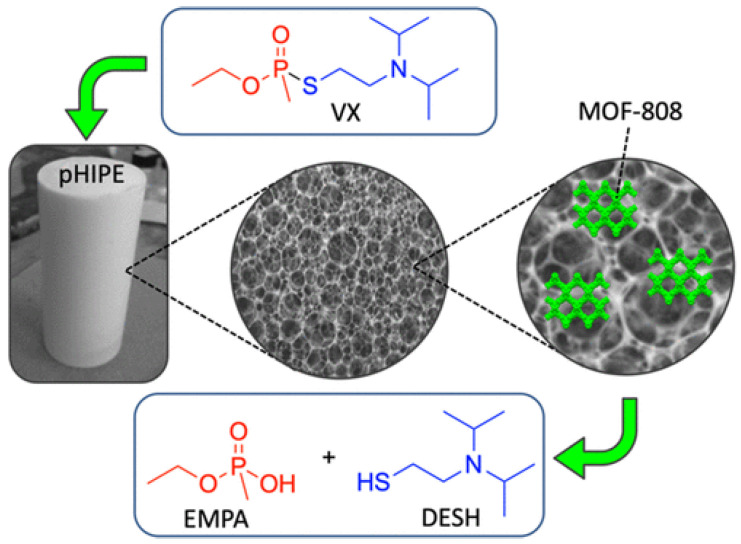
The structure of the MOF−HIPE composite and the degradation of VX. Reprinted with permission from [115]. Copyright 2020 American Chemical Society.

A metal−organic framework-containing polymer sponge was fabricated by combining the excellent nerve agent absorption agent (styrene pHIPE) with MOF-88, which served as a hydrolysis catalyst (Figure 15) [115]. This MOF-HIPE composite can facilitate the bulk absorption, immobilization, and catalytic decomposition of P–S bonds in VX, and they rapidly hydrolyze over 80% VX in 8 h with a half-life of less than 1 h. The fibrous MOF-808 nanozyme aerogel, which was fabricated by in situ growth of MOF-808(Zr-MOF) on cellulose nanofibers, has a hierarchical macro/microporosity that provides more accessible active sites. This flexible and processable monolithic MOF composite aerogel demonstrated superior catalytic performance for hydrolysis with a very short half-life of 1 min, and DMNP was converted to nontoxic dimethyl phosphate (DMP) [112]. Superelastic cellular hierarchical metal–organic framework aerogels can be fabricated by combining functional MOFs-88 nanoparticles with structural SiO_2_ nanofibers based on hydrogen bond-assisted interfacial coupling effect. The as-prepared MOF-808/SiO_2_ aerogels have a preserved MOF structure, van der Waals barrier channels and minimized diffusion resistance, which all contribute to increasing the adsorption and decontamination efficiency toward CWAs. This optimized aerogel-based MOF exhibited rapid adsorption and detoxification properties for DMMP with a half-life of 5.29 min [104].

In addition, a hydrogel with polymeric networks, mechanical stability, flexibility and a high water content is a very suitable platform for the hydrolytic reaction of nerve agents, and many MOFs/hydrogel composites have been reported. For example, the inexpensive non-volatile branched polyethyleneimine hydrogel integrated with Zr-MOFs was developed for rapid degradation of organophosphorus chemicals [108]. The hydrogel possesses high amine density and plentiful water, which can regulate the micro-environment of the MOF catalytic reaction process. The obtained MOF-808 hydrogel (MOF-808/BPEIH) powder can induce near-instantaneous catalytic hydrolysis of DMNP with a short initial half-life of less than 1 min under ambient humidity, which is better than all other reported MOF-based composites. When the MOF-based composite was coated onto a textile, the as-prepared MOF-808/BPEIH/fiber composite also possessed excellent catalytic activity for DMNP with an initial half-life of 1 min and a conversion of 72% after 15 min. Regarding actual nerve agents, VX and GD, the MOF-808/BPEIH/fiber composite can degrade nearly all VX and nearly 60% of the GD after 10 min under ambient conditions, demonstrating potential for the large-scale production of protective gear in practical conditions.

The SA@UiO-66-NH_2_@PAMAM composite hydrogel synthesized by immobilizing UiO-66-NH_2_ and PAMAM to the backbone of sodium alginate can rapidly degrade DMNP with a half-life as short as 7 min. This composite hydrogel easily combines with cotton fabric. Upon introducing the indicator 4-nitro-(dimethyl-tert-butyl) silica ether (P-NSE), the obtained recyclable flexible cotton not only catalyzes the hydrolysis of the nerve agent GB but also serves as a portable colorimetric platform to realize the real-time visual detection of changes in degradation [114].

To explore efficient catalysts for the destruction of nerve agents under atmospheric environments, a spontaneously super-hygroscopic MOF-gel microreactor was designed and synthesize by photoinduced integration of UiO-66-acrylamide (UiO-66-AM) and alkaline poly(dimethylaminoethyl acrylate) onto LiCl-salinized poly(N-isopropylacrylamide) gel. The resultant MOF@PDMAEA@LiCl@PNIPAM gel (MG) exhibits excellent catalytic performance for hydrolysis of DMNP with an initial half-life of ~1.9 h, and the final conversion is 95.5% [111].

### 5.2. Vesicant Agents and Simulants

**Figure 16:**
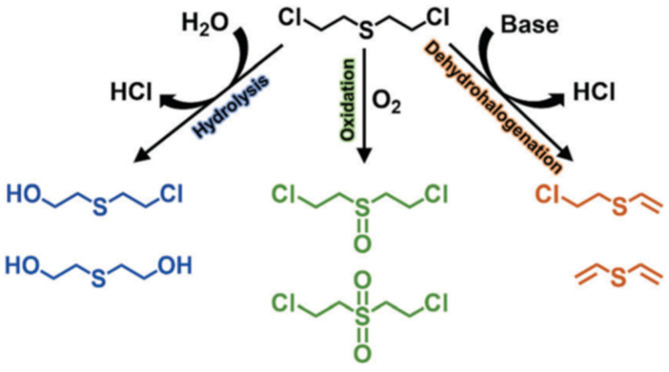
HD degradation pathway. Reproduced from Ref. [25] with permission from the Royal Society of Chemistry.

Sulfur mustard (HD), which was first used in World War I, remains the most notorious vesicant agent. The degradation process of HD includes oxidation, dehalogenation, and hydrolysis (Figure 16), and the C–Cl of HD will be destroyed during the hydrolysis process [25]. CEES is commonly used in experiments instead of HD given its high toxicity. At present, there are many studies that focus on the detoxification of HD or simulants, which all exhibit remarkable potential in future military applications.

Pure monolithic UiO-66-NH_2_ xerogel reported by Zhou and our group also demonstrated a fast decomposition rate of 2-CEES with a half-life of 8.2 min, higher than that of UiO-66-NH_2_ powder (t_1/2_ = 29 min) [105]. Further study showed this xerogel has a t_1/2_ value of 14.4 min for the hydrolytic degradation of the real CWA sulfur mustard (HD).

**Figure 17:**
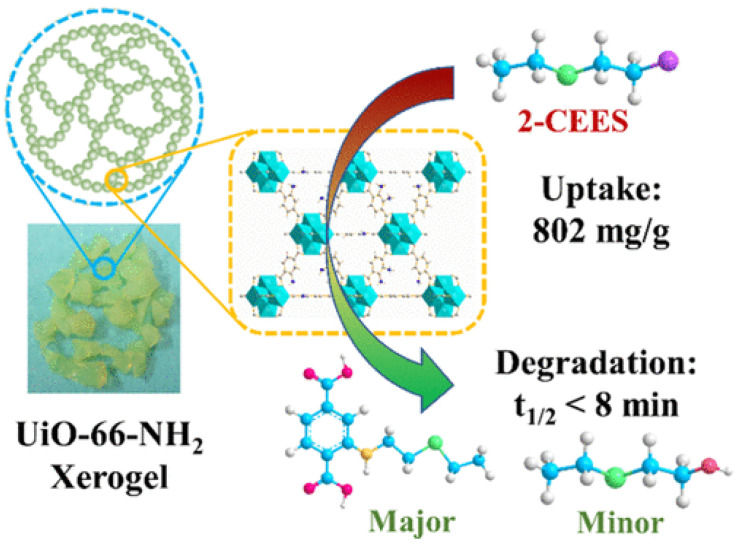
Structurally defective granular UiO-66-NH_2_ xerogels and the hydrolysis of 2-CEES. Reprinted with permission from [103]. Copyright 2022 American Chemical Society.

Together with our collaborators, we designed and synthesized a series of defective granular UiO-66-NH_2_ xerogels and investigated their catalytic properties for the decontamination of 2-chloroethyl ethyl sulfide (2-CEES) (Figure 17) [103]. The degradation rate increased with the increasing defect degrees and reducing the size of MOF crystals. A shortened half-life value of 7.6 min was observed, representing the best performance for MOFs reported under ambient conditions [103].

By combining UiO-66-NH_2_ and aramid nanofibers (ANFs), a light weight, flexible, and mechanical robust aerogel with a 3D hierarchically porous architecture was constructed. The resultant UiO-66-NH_2_@ANF aerogels have a short half-life of 8.15 min for the detoxification of 2-chloroethyl ethyl thioether (CEES), and the removal rate is as high as 98.9%. The C-Cl bond in CEES was broken, and the fragment recombined to form BETE with low toxicity. This aerogel exhibits good mechanical stability with a recovery rate of 93.3% after 100 cycles [109].

In practical application scenarios, multiple chemical warfare agents may be used at the same time, so the ability of MOF gel to correspond to multiple toxic substances at the same time should also be explored. The monolithic UiO-66-NH_2_ xerogel has initially demonstrated this capability and may have important application prospects.

## 6. Conclusions and Outlook

In summary, many studies demonstrate the potential of MOF gels and their composites as effective materials for the detoxification of chemical warfare agents (CWAs). The unique properties of MOF gels, such as their large specific surface area, hierarchical porous structures, and processability, make them highly suitable for this application. The studies have focused on nerve agents and vesicant agents, with Zr-MOF gels being the most commonly reported material.

Various approaches have been explored to enhance the catalytic performance of MOF gels, including the development of pure MOF gels, MOF-loaded composites, and MOF/hydrogel composites. The results have shown rapid and efficient degradation of CWAs, with short half-lives and high conversion rates achieved within minutes. The use of flexible and processable monolithic MOF composite aerogels has further improved the catalytic performance, enabling the disposal of significant quantities of CWAs per hour.

In addition, the combination of MOFs with different matrices, such as cellulose, graphene, and balsa wood, has expanded the functionalities and advantages of MOF gels. Silica aerogel-based MOF composites have demonstrated low densities and high specific surface areas, while wood-based aerogels have shown potential for cost-effective and continuous production. These advancements pave the way for future military applications of MOF gels in CWA detoxification.

Looking ahead, further research should focus on optimizing the performance and stability of MOF gels, exploring the potential of other MOF compositions, and investigating their efficacy against a broader range of CWAs. The coupling effects of other external conditions, such as light, microwave, ultrasound, and piezoelectric conditions, on the adsorption and catalytic degradation process should be studied, which will provide new ideas for developing efficient protecting materials. Additionally, efforts should be made to scale up the production processes and evaluate the feasibility of incorporating MOF gels into practical systems for large-scale CWA decontamination. By continuing to explore and refine the application of MOF gels for CWA detoxification, significant advancements can be made in the fields of chemical defense and military protection.

## Figures and Tables

**Table 1:** Various MOF gels Used as Protection Media for the Detoxification of CWAs.

Materials	Amount or Size	Agent Volume	Half-Life	Environment	Mechanism	Refs.
monolithic UiO-66 xerogel	25 mg	HD, 2.5 μL	24.8 min	Liquid-Phase	Catalytic hydrolysis	[105]
monolithic UiO-66 xerogel; monolithic UiO-66-NH_2_ xerogel	20 mg	VX, 0.4 μL	≤1.5 min	Liquid-Phase	Catalytic hydrolysis	[105]
monolithic UiO-66-NH_2_ xerogel	25 mg 25 mg	HD, 2.5 μL 2-CEES, 2.5 μL	14.4 min 8.2 min	Liquid-Phase	Catalytic hydrolysis	[105]
granular UiO-66-NH_2_ xerogel	10 mg	2-CEES, 1 μL	7.6 min	Liquid-Phase	Catalytic hydrolysis	[103]
UiO-66-NH_2_-loaded cellulose sponge	8.1 mg	DMNP, 4 μL	9 min	Liquid-Phase	Catalytic hydrolysis	[106]
UiO-66/Nanocellulose Aerogel	8 mg	MPO, 2.5 μmol	0.7 min	Liquid-Phase	Catalytic hydrolysis	[107]
MOF-808/BPEIH hydrogel	2.2 mg MOF-808 loading, 6 mol%	DMNP, 4 μL	<1 min	Liquid-Phase	Catalytic hydrolysis	[108]
MOF-808/BPEIH/fiber	1 × 1 cm, containing 1.5 μmol MOF-808	DMNP, 4 μL GD, 3 μL DEMP, 4.2 μL	1 min <10 min <1 min	Liquid-Phase	Catalytic hydrolysis	[108]
MOF-808/SiO_2_ aerogels	200 mg	DMMP, 4 μL	5.29 min	Liquid-Phase	Catalytic hydrolysis	[104]
UiO-66-NH_2_@ANF aerogels	20 mg	CEES, 5 μL	8.15 min	Liquid-Phase	Catalytic hydrolysis	[109]
UiO-66-NH_2_@agarose hydrogels	—	DCP, vapors	—	atmospheric	Catalytic hydrolysis	[110]
UiO-66-AM @PDMAEA@LiCl@PNIPAM aerogel	60 mg of UiO-66-AM loading, 12 mol%	DMNP, 12.5 μmol	1.9 h	atmospheric	Catalytic hydrolysis	[111]
fibrous MOF-808 nanozyme aerogel	1 mm × 1 mm × 1 mm, containing 1.5 μmol MOF-808	DMNP, 25 μmol	1 min	Liquid-Phase	Catalytic hydrolysis	[112]
MOF-808/bacterial cellulose sponge	1.5 μmol MOF-808 in composite	DMNP, 25 μmol	<1 min	Liquid-Phase	Catalytic hydrolysis	[113]
SA@UiO-66-NH_2_@PAMAM hydrogel	17.6 mg	DMNP, 4 μL	7 min	Liquid-Phase	Catalytic hydrolysis	[114]
MOF-808/HIPE sponge	3.2 mg MOF-808 loading, 0.68 mol%	VX, 24 µL	<1 h	Liquid-Phase	Catalytic hydrolysis	[115]
UiO-66/DSPD Composite Films	1 × 1 × 0.015 cm^3^	MPO, 25 μmol	—	Liquid-Phase	Catalytic hydrolysis	[116]

“—” = not mentioned.
